# Mental health and COVID-19 in South Africa

**DOI:** 10.1177/00812463211001543

**Published:** 2021-06

**Authors:** Siphelele Nguse, Douglas Wassenaar

**Affiliations:** College of Humanities, University of KwaZulu-Natal, South Africa

**Keywords:** COVID-19, gender-based violence, mental health, poverty, psychology

## Abstract

COVID-19, the disease caused by severe acute respiratory syndrome coronavirus 2, has affected most parts of the globe since its first appearance in the city of Wuhan, China, in December 2019. As a result, the World Health Organization declared the virus a global public health crisis and a pandemic within 2 weeks, after the virus had spread to 114 countries with 118 000 recorded cases and 4291 deaths due to the virus and related complications. The World Health Organization declaration is indicative of the enormous impact of the pandemic on human life globally. South Africa has not been exempted from that impact. While the pandemic has affected all South Africans in various ways, the poor have been most affected due to structural inequality, poverty, unemployment, and lack of access to quality health care and other services. Furthermore, public mental health has also been negatively affected by the pandemic, and this comes against a backdrop of an ailing mental health care system. We argue that the psychology profession, as a mental health profession and behavioural science, working as part of a multidisciplinary team, ought to play a significant role in addressing the mental health ramifications of the pandemic. In so doing, lessons can be drawn from other countries while establishing contextual immediate and long-term interventions.

Since 5 March 2020, when the Minister of Health confirmed the first South African positive test of COVID-19, the virus has become an integral and unfortunate part of everyday life in South Africa. Daily bulletins reporting current incidence and mortality remind each citizen of the ongoing threat of the virus and its potential life-changing complications. These include statistics about persons who have either tested positive for the virus, died due to complications related to it, or recovered from it. The virus comes against a backdrop of a protracted public health crisis in South Africa – the mental health gap (Burns, 2011; Marais & Petersen, 2015; October, 2019; Pillay, 2017; WHO, 2007). The pandemic has amplified the existing mental health gap and constrained access to mental health care services. This article aims to explore the state of mental health and illness in South Africa and the local and global mental health impact of the coronavirus pandemic. In so doing, the article will outline the dynamic and intersectional effects of the virus on the well-being of South Africans, particularly the poor, who, due to structural inequality, poverty, unemployment, and other socioeconomic constraints, find themselves marginalised in terms of access to quality health care (Harris et al., 2011; South African Human Rights Commission [SAHRC], 2019; Statistics South Africa, 2020; Tomita et al., 2020). Finally, the article makes recommendations to remedy the neglect of mental health care in dealing with the pandemic.

## The state of mental health in South Africa

Mental health has been treated as a peripheral and insignificant part of the health sector (Burns, 2011; October, 2019; Pillay, 2019). This has been to the detriment of those who live with mental illnesses, undermining and limiting access and quality of mental health care services. This is reflected in the paucity of investment and limited government contribution to mental health (Docrat et al., 2019; October, 2019). The South African government may be applauded for the legislative and policy direction it has taken in prioritising access to mental health care. Legislation and policies such as the Mental Health Care Act of 2002 and the National Mental Health Policy Framework and Strategic Plan 2013–2020 are evidence of such direction (Republic of South Africa, 2002, 2013); however, there exists a gap between policy development and implementation. The lack of a sustainable funding model for mental health perpetuates the lack of mental health services, as there is no clear source of funding for the implementation of the aforementioned policies and plans. Furthermore, while South Africa, compared to other African countries, has shown some improvements in the legislation and provision of mental health care services, the ratio of physical/biological to psychiatric/psychological health care remains unbalanced (Marais & Petersen, 2015; Petersen et al., 2017). Burns (2011) notes this gap between physical and mental health services and refers to it as a violation of human rights. Indeed, the treatment of mental health in South Africa is a case for violation of human rights and disregard for the lives of those who live with mental illnesses. It borders on ableism and a lack of empathy for mentally ill people. Marais et al. (2020) state that the inadequacy and paucity of mental health care services may reflect (1) exclusion of mental health practitioners and service-users from the consultative process leading to the development of policies, (2) limitations in the implementation of experiential knowledge-informed policies. Furthermore, the mental health care service gap in South Africa has been well documented in other policy proposals and academic research (Burns, 2011; October, 2019; Pillay, 2019; Pillay & Barnes, 2020; SAHRC, 2019; WHO, 2007). Data presented by the South African College of Applied Psychology (2018) suggest that one in six South Africans suffers from anxiety, depression, or a substance use disorder, 40% of South Africans living with HIV have a comorbid mental disorder, 41% of pregnant women are depressed, about 60% of South Africans could be suffering from post-traumatic stress – this figure includes motor vehicle accidents and crime, while only 27% of South Africans with severe mental disorders receive treatment. The fact that only 27% of those living with severe mental disorders receive treatment is an indication of how mental illness has been neglected by the health system. Numerous other examples demonstrate the magnitude and severity of the state of mental health care in South Africa. October (2019), for example, reports that mental health is allocated 5% of the national health budget, while only 50% of public hospitals offering mental health services have a psychiatrist, and about 30% do not have a clinical psychologist. The abhorrent Life Esidimeni incident tragically illustrates the neglect of mental health services in South Africa (Dhai, 2017; Makgoba, 2017; Pillay, 2019; SAHRC, 2019). October (2019) reported that 144 mental health care users died in this incident, nine had not been found by 2019, 4 years after the negligent movement, and ‘treatment’, of mental health care users referred from a state facility to unregistered and unsuitable nongovernmental hospitals (NGOs). The Life Esidimeni incident was an avoidable tragedy but was consistent with the way mental health had, and remains, been treated as a peripheral and insignificant element of public health care. The question that remains is what lessons have been learned from the incident? What measures and developments have been put in place to prevent the repetition of such a brutal and inhumane incident? What have the government and the legal system done about those who were found to have been responsible for the approval of the transfer of patients to unregistered and unequipped NGOs and the other irregularities contained in the Makgoba (2017) report? These questions take us to the second part of this article, which explores the impact of COVID-19 on the state of mental health in South Africa, considering the inadequate state of mental health care services and facilities that preceded COVID-19.

## COVID-19 and mental health in South Africa

COVID-19 has had a significant impact on mental health (A. W. Kim et al., 2020; Naidu, 2020; Pillay & Barnes, 2020). A. W. Kim et al. (2020) in their recent study of the mental health impact of COVID-19 on South Africans living in Soweto, found that adults who had experienced childhood trauma and other related adversities, were at higher risk of developing depressive symptoms precipitated by the perceived risk of contracting COVID-19. In addition, Naidu (2020) states that due to public biological, psychological, and social predispositions in South Africa, COVID-19 may lead to mental health presentations such as post-traumatic stress disorder, mood disorders, anxiety disorders, phobias, and obsessive-compulsive disorders. A study conducted by the Human Sciences Research Council (2020) reported that 33% of South Africans were depressed, while 45% were fearful, and 29% were experiencing loneliness during the first lockdown period. While the provision of and access to essential services, including mental health care, was permitted during the lockdown period, as gazetted by Government (Lockdown regulations, No.: 43232, April 2020), some mental health care users were unable to access services due to limitations and risks presented by physical contact and in-person consultations (Pillay & Barnes, 2020). Furthermore, Govender (2020), a psychologist working for *Doctors Without Borders*, an NGO providing health support and services in different countries – including South Africa – reported a decrease in mental health visits during the lockdown period in one of their facilities in Tshwane. From personal observation in clinical practice, the first author noted that the number of patients who defaulted their psychotherapy appointments was higher than usual during the initial stages of the lockdown period. Some of the reasons cited by patients were fear and anxiety about contracting the virus, transport difficulties, retrenchment, and unemployment. Furthermore, and anecdotally, several patients who attended their psychotherapy sessions reported secondary impacts of the coronavirus pandemic, such as sleep disturbance, anxiety, depressive symptoms, unemployment, food insecurity, substance withdrawal symptoms, and intensified abuse in their homes. The mental health care challenges were not only limited to outpatients but were also applicable to inpatients. Although there is no recorded data on patient incidents among patients in state/provincial long-term care facilities during the lockdown period, personal observation during clinical practice suggested that there was an increase in patient incident reports for various reasons, including irritability resulting from the inability to access tobacco due to the lockdown regulations, discontinuation of patient visits and leaves of absence, and psychological distress.

While this article focuses on the mental health impact of COVID-19, it is important to note that service uptake decline was not limited to mental health, but to the broad health spectrum. Siedner et al. (2020), in their study on access to primary health care in rural KZN, found a decrease in child health care and HIV-related visits during the lockdown period. Furthermore, Joska et al. (2020) reported an increased risk to mental health and safety for women living with HIV due to COVID-19 and lockdown regulations. The Right to Care (2020), in a media statement, reported a major decrease in hospital visits for people living with HIV and other chronic illnesses. Msomi (2020) reported that socioeconomic factors such as transport, unemployment, and food insecurity constrain capacity to attend routine hospital and outpatient visits. These have been exacerbated by the lockdown period. Approximately 3 million South Africans lost their employment during the first 4 months of the lockdown period (Ingle et al. 2020). This major increase in unemployment comes on top of already alarming national unemployment data. Statistics South Africa (2020) reported that South African unemployment had risen to 30.1%. Furthermore, on 2 April 2020, the Minister of Police stated that 87,000 cases of gender-based violence (GBV) were reported nationally during the first week of the lockdown period (Chothia, 2020). Research suggests that only a minority of GBV cases are reported for various reasons, including incompetent and unjust handling of GBV cases in the justice system, abuse and harassment of survivors by perpetrators and cultural and socioeconomic dynamics (Centre for Study of Violence and Reconciliation, 2016; Middleton, 2011; Mpani & Nsibande, 2015; South African Human Rights Commission., 2018).

The above review suggests that there has been significant suffering by many South Africans during the lockdown period, considering also that much goes unreported and undetected. Additional challenges have been posed by how the government has dealt with care for persons diagnosed with COVID-19. Resources and energy have been concentrated on the biomedical aspect of the virus with relative disregard for the mental health impact of the virus. Singh (2020a) in his critique of government’s management of the virus, states that four areas of knowledge (pathologists and laboratory, clinicians, public health experts; and researchers) have been prioritised to the exclusion of other equally significant areas of psychosocially relevant expertise such as anthropology and religion, political and behavioural sciences, law and economics. In addition, Bank (2020), drawing lessons from the Ebola crisis, warns governments to guard against neglecting behavioural and socioeconomic aspects of the virus in the same way that countries in the Global North and Western Europe did, as this may have negative effects in combatting the virus. Government’s prioritisation of biomedical interventions is evident in the 8-stage response to the pandemic as reported by the Department of Health’s Ministerial Advisory Committee (Singh, 2020b). According to the 8-stage response plan, the management of the psychological and social impact of the pandemic is in the seventh stage. If this response is to be implemented as is, the devastating impact of the virus would be felt mostly by mental health care users and professionals. Perhaps in response to this, there is now also a Socio-Behavioural Ministerial Advisory Committee on COVID-19, effective from October 2020, implicitly recognising data from the ‘first wave’ indicating that the mental health impact of the pandemic would have further social and economic effects. As indicated above, many South Africans are already experiencing the mental health impact of the pandemic. This has been compounded by adverse socioeconomic factors such as unemployment, poverty, lack of access to clean water and sanitation, GBV, inadequate housing, and other factors (Chothia, 2020; Human Sciences Research Council, 2020; Msomi, 2020; Orkin et al., 2020; Pillay & Barnes, 2020; Statistics South Africa, 2020).

## Global mental health impact of COVID-19

The mental health impact of COVID-19 is also a global crisis. Although it remains an ongoing discussion, lessons can be drawn from the experiences of other countries to build contextual responses. Ornell et al. (2020) argue that mental health is usually ignored and disregarded in times of infections and pandemics, yet the mental health and socioeconomic ramifications may be more lasting than the overall physiological impact of the infection for the average citizen. The global coronavirus pandemic is not immune to such impact. The aforementioned mental health ramifications of the pandemic have resulted in engagements and proposals on mitigation strategies, programmes, and policies (Torales et al. 2020). Dong and Bouey (2020) reported on China’s response, which included the establishment of nationwide mental health response measures and services, though the authors raised concerns about the lack of detailed communication from government. This strategy by the Chinese government and the National Health Commission of China, despite the shortfalls described by the authors, recognises the mental health impact of the pandemic. In addition, Marazziti (2020) and Marazziti and Stahl (2020), writing from an Italian context, call for mental health components in coronavirus interventions. Their recommendations include support for frontline health care workers and other professionals and the implementation of nationwide mental health care services to assist in dealing with the coronavirus-related mental health complications for individuals and communities. Heale and Wray (2020) call for support, care, awareness, and investment of resources towards mental health care workers and users in response to COVID-19. In the United States, the coronavirus, as in all other unequal societies, has had a relatively greater adverse mental health impact on disenfranchised and marginalised black Americans (WHO, 2020). Novacek et al. (2020) therefore argue that governments must implement responsive measures aimed at addressing the mental health effects of the pandemic on black Americans. In addition, Breslau et al. (2020), reporting on a nationwide longitudinal study assessing the mental health impact of the coronavirus pandemic in the United States, found a correlation between prepandemic psychological distress, economic distress, unemployment, ethnicity, and gender. They found that those with pre-existing psychological distress, Hispanics, unemployed people, women, and those working in stressful work environments had serious psychological distress during the first month of the pandemic. The psychological distress that was observed in the first month of the pandemic was in line with prepandemic psychological distress among the aforementioned groups. What is evident from the above United States reports is that the mental health ramifications of the coronavirus, while felt by all sectors of society, has a more adverse impact on marginalised population groups compared to the rest of society, due to pre-existing socioeconomic conditions that are exacerbated by the pandemic. Holmes et al. (2020) call for a multidisciplinary approach in dealing with the global coronavirus pandemic. They argue that a multidisciplinary approach is essential for dealing with the physical, psychological, social, and neurological impacts of the virus, particularly on vulnerable populations. In line with the multidisciplinary approach, Ince and Minhas (2020), drawing lessons from the coronavirus pandemic, outline the importance of including disaster management in the training of health professionals in preparation for future health disasters. Accordingly, Banerjee and Nair (2020) proposed a 7-step community-based tool kit to assist in dealing with the public and mental health impact of the coronavirus pandemic. These include (1) the collection of basic information, (2) crisis management, (3) open and inclusive communication, (4) individual care, (5) family support and care, (6) community-based care and interventions, and (7) organisational restructuring, adjustment, and support. Furthermore, Grover et al. (2020) call for the inclusion and deployment of mental health professionals in all COVID-19 hospitals and facilities.

## The role of psychology in response to the mental health impact of COVID-19 in South Africa

The local and global mental health impact of COVID-19 clearly requires a focused and assertive response by mental health workers. Psychology, as a mental health profession and behavioural science, ought to be at the forefront of this task. Clear evidence of the mental health impact of the coronavirus pandemic should be included in the policy framework and government strategies, not as an incidental issue, but as an integral part of the nationwide plan, with a clear focus on vulnerable individuals and communities. It is encouraging to note that there are now several psychologists on the revised (October 2020) Ministerial Advisory Committee on COVID-19. Prior to this, Pillay and Barnes (2020) noted the *ad hoc* commitment and involvement of some psychologists and other professionals in highlighting and contributing to the mental health impact of the coronavirus. They have further called for a critical, socially responsive, and engaged psychology in response to the coronavirus pandemic. Formal psychology organisations such as the *Psychological Society of South Africa* (PsySSA, 2020c), have developed and shared resources to aid practitioners’ response to the pandemic and its mental health ramifications. In addition, PsySSA (2020a; 2020b) has launched various interventional services such as the Health Workers Network, a voluntary service appealing to registered psychologists to offer *pro deo* short-term telephonic counselling to schools, and partnered with the Independent Community Pharmacy Association (ICPA) to offer voluntary services to victims and survivors of GBV. Other organisations, such as the South African Anxiety and Depression Group (SADAG), and individuals have also offered virtual support to health care workers, schools, and community members. All these initiatives are commendable because they symbolise critical elements of a professional contribution to the COVID-19 pandemic that is research-informed, evidence-based, and socially responsive.

The mental health ramifications of the coronavirus pandemic are likely to have a lasting impact on many South Africans. As emphasised above, this comes against the backdrop of an already ailing health system and paucity of mental health services. For this reason, mental health care must be included in government interventions and plans. The psychology profession, as a mental health profession and behavioural science, ought to lead and contribute actively to this effort, as stated above. The contribution of the profession should also extend to the training of psychologists and mental health professionals in immediate and responsive treatment measures such as Psychological First Aid (Bymer et al., 2006), Solution-Focused Brief Psychotherapy (J. S. Kim, 2013), and longer-lasting treatment models in preparation for the documented possible mental health sequelae of the virus. Taking the above circumstances and priorities into account, we argue here that South African professional psychology training programmes need to revisit and expand their programmes to include systematic public health perspectives and applications, to complement the individual and community perspectives already widely taught (Eaton & Fallin, 2019). In addition, the Health Emergency and Disaster Risk Management Framework (WHO, 2019) ought to be included in the training of psychologists to equip professionals in dealing with health disasters. This framework includes mitigation, preparation, response, recovery and prevention. Furthermore, a multidisciplinary approach must be employed by the government and all health professionals in dealing with the pandemic. Inpatient and outpatient mental health care and treatment should be reviewed and repurposed by mental health care professionals to maximise treatments for the reported psychosocial impacts of the coronavirus. The impact of the lockdown regulations on the delivery and access to mental health care services ought to be considered by all mental health care professionals. Resources should be invested in alternative intervention modalities if the adverse psychological and social effects of the virus and lockdown measures are to be reduced as much as possible to maximise the country’s social recovery once an effective vaccine, and other efficacious prevention and treatment measures, are in place.

## Conclusion

This article has outlined evidence highlighting current and likely future mental health ramifications of the coronavirus pandemic in South Africa. The pandemic came against the backdrop of an ailing, unprepared, and inadequate health care system characterised by a paucity of mental health services, among other resource constraints and challenges. Furthermore, this article demonstrates that while the coronavirus pandemic affects all South Africans, the poor and marginalised are the most affected due to socioeconomic realities such as inequality, poverty, GBV, and rising unemployment. In addition, we argue that the government’s response to the pandemic should not be focused on the biomedical aspects of the virus to the exclusion of other equally significant strategies and plans, offered by other disciplines. We are heartened to note the expanded disciplinary spectrum on the revised Ministerial Advisory Committees on COVID-19 implemented in October 2020. Furthermore, a national survey to assess access to mental health care services during the lockdown period is warranted. Finally, the paper calls for action and advocacy from the psychology profession in ensuring that the mental health ramifications of the pandemic are considered in the development of response strategies and plans in dealing with the pandemic and its’ impact on the lives of South Africans.
